# Relationship between daylength and suicide in Finland

**DOI:** 10.1186/1740-3391-9-10

**Published:** 2011-09-23

**Authors:** Laura Hiltunen, Kirsi Suominen, Jouko Lönnqvist, Timo Partonen

**Affiliations:** 1 National Institute for Health and Welfare, Department of Mental Health and Substance Abuse Services, P.O. Box 30 (Mannerheimintie 166), FI-00271 Helsinki, Finland; 2 Helsinki University Central Hospital, Jorvi Hospital, Department of Psychiatry, Espoo, Finland; 3 University of Helsinki, Department of Psychiatry, Helsinki, Finland

**Keywords:** circadian clock, suicide, light-dark transition, sunshine, temperature

## Abstract

**Background:**

Many previous studies have documented seasonal variation in suicides globally. We re-assessed the seasonal variation of suicides in Finland and tried to relate it to the seasonal variation in daylength and ambient temperature and in the discrepancy between local time and solar time.

**Methods:**

The daily data of all suicides from 1969 to 2003 in Finland (N = 43,393) were available. The calendar year was divided into twelve periods according to the length of daylight and the routinely changing time difference between sun time and official time. The daily mean of suicide mortality was calculated for each of these periods and the 95% confidence intervals of the daily means were used to evaluate the statistical significance of the means. In addition, daily changes in sunshine hours and mean temperature were compared to the daily means of suicide mortality in two locations during these afore mentioned periods.

**Results:**

A significant peak of the daily mean value of suicide mortality occurred in Finland between May 15th and July 25th, a period that lies symmetrically around the solstice. Concerning the suicide mortality among men in the northern location (Oulu), the peak was postponed as compared with the southern location (Helsinki). The daily variation in temperature or in sunshine did not have significant association with suicide mortality in these two locations.

**Conclusions:**

The period with the longest length of the day associated with the increased suicide mortality. Furthermore, since the peak of suicide mortality seems to manifest later during the year in the north, some other physical or biological signals, besides the variation in daylight, may be involved. In order to have novel means for suicide prevention, the assessment of susceptibility to the circadian misalignment might help.

## Background

Current data on the routinely occurring peaks of deaths from suicide are conflicting [1,2]. However, for the past four decades in Finland, the seasonal pattern has been stronger the lower the suicide mortality has been [3]. There is a clear peak of suicide occurrence around May or June [4-7] and a preceding peak in suicide attempts around April [8]. Furthermore, another smaller peak of suicide occurrence exists around October [7,9]. These two mortality peaks, being similar and more robust the further away the country locates from the equator, have been explained by socio-demographic and socio-economic factors [10], but since this seasonal pattern has existed for decades [11], if not centuries [12], biological factors are likely.

Major depressive episodes are known to contribute to suicide substantially [13,14], and a history of mood disorders and psychiatric hospitalization associates clearly with the seasonal occurrence of suicides [15,16]. Desynchronization of physiological rhythms, e.g. desynchronization of the circadian rhythm of core body temperature with the sleep-wake cycle [17-19] and some clock gene variants [20,21], can be associated with mood disorders. Based on our earlier psychological autopsy studies of death from suicide [22] and the data from the nationwide suicide program in Finland [23], we hypothesized that the circadian misalignment among the depressed may increase during spring, and thereby predispose to suicidal behaviors [24].

Rest-activity cycles during the day [25] and sleep stages at night [26] are controlled by circadian clocks, but they are frequently disturbed among the depressed. Furthermore, the principal circadian clock entrains to the sun light [27-29], by tracking the daily changes in rise and set times of the sun and the variation in the length of the day [30-32]. Thus, the timing of light exposure is relevant to entrainment and influences the course of mood disorders [33,34]. Therefore, we hypothesized that it is the key to the suicide mortality peaks whether the light-dark transitions give the principal circadian pacemaker a signal to accelerate or decelerate, especially among the depressed. In addition, since sunshine and ambient temperature are potential time-givers, modulate the function of biological clocks [35], and associate with deaths from suicide [3,36], we aimed to test their effect, as well.

## Methods

Statistics Finland http://www.stat.fi provided us with the daily data of 43,393 suicides, 33,993 of men and 9400 (22%) of women, committed in Finland during the 35-year period of 1969 to 2003 (Tables 1 and 2). Two phenomena, which affect the timing and the speed of the light-dark transitions regularly each year, were selected a priori as the potential factors that might challenge the biological clocks and produce circadian misalignment. First, we focused on the length of the photoperiod, because at high to temperate latitudes around spring and fall equinoxes the transitions between day and night are most rapid and the durations of twilight short, as a consequence of the rotation of the earth. Second, we focused on the constant mismatch between the sun time (hereafter ST) and the coordinated universal time (hereafter UCT), arising from the earth's tilt and elliptical orbit around the sun.

**Table 1:** Men's suicides in numbers during the study period

	*Oulu*			*Helsinki*			*Finland*		
**Year**	**Men**	**S**	**per** **100** **000**	**Men**	**S**	**per** **100** **000**	**Men**	**S**	**per** **100** **000**

1969	42 447	11	25.92	272 321	110	40.39	2 230 217	850	38.11
1970	41 412	13	31.39	266 174	108	40.58	2 219 985	763	34.37
1971	42 798	11	25.70	271 393	117	43.11	2 234 037	781	34.96
1972	43 436	22	50.65	275 378	132	47.93	2 249 051	874	38.86
1973	44 127	27	61.19	277 205	109	39.32	2 262 142	849	37.53
1974	45 082	22	48.80	278 485	131	47.04	2 273 815	921	40.51
1975	45 815	22	48.02	278 628	128	45.40	2 282 115	924	40.49
1976	46 069	29	62.95	278 693	152	54.54	2 286 392	967	42.29
1977	46 444	22	47.37	277 978	154	55.40	2 295 668	962	41.91
1978	46 609	13	27.89	277 735	156	56.17	2 300 790	963	41.86
1979	46 533	18	38.68	278 569	133	47.74	2 306 784	935	40.53
1980	46 779	24	51.31	279 456	145	51.89	2 314 843	962	41.56
1981	47 343	21	44.36	280 580	151	53.82	2 327 473	904	38.84
1982	48 179	18	37.36	282 751	134	47.39	2 342 869	905	38.63
1983	48 331	25	51.73	284 565	130	45.68	2 357 172	938	39.79
1984	48 620	25	51.42	286 092	149	52.08	2 369 228	988	41.70
1985	49 065	23	46.88	287 858	113	39.26	2 377 780	964	40.54
1986	49 405	30	60.72	290 370	149	51.31	2 385 866	1023	42.88
1987	49 890	28	56.12	292 935	137	46.77	2 392 868	1068	44.63
1988	50 138	44	87.76	294 242	150	50.98	2 401 368	1112	46.31
1989	50 951	29	56.92	295 665	160	54.12	2 412 760	1121	46.46
1990	51 623	33	63.93	298 420	198	66.35	2 426 204	1199	49.42
1991	52 254	35	66.98	302 609	185	61.14	2 443 042	1193	48.83
1992	52 959	36	67.98	306 298	204	66.60	2 457 282	1160	47.21
1993	53 495	35	65.43	311 134	172	55.28	2 470 196	1112	45.02
1994	54 661	23	42.08	316 367	176	55.63	2 481 649	1080	43.52
1995	56 132	26	46.32	322 074	179	55.58	2 491 701	1081	43.38
1996	57 436	26	45.27	327 168	131	40.04	2 500 596	966	38.63
1997	58 482	36	61.56	332 113	158	47.57	2 509 098	1039	41.41
1998	59 606	26	43.62	337 297	121	35.87	2 516 075	965	38.35
1999	61 025	40	65.55	341 125	139	40.75	2 523 026	961	38.09
2000	62 800	28	44.59	344 520	143	41.51	2 529 341	879	34.75
2001	64 116	31	48.35	347 925	150	43.11	2 537 597	936	36.89
2002	64 995	22	33.85	349 121	139	39.81	2 544 916	825	32.42
2003	65 965	29	43.96	350 334	119	33.97	2 552 893	823	32.24

The yearly male population, number of suicides(S), and suicide mortality for men in Oulu, Helsinki, and Finland from 1969 to 2003.

**Table 2:** Women's suicides in numbers during the study period

	*Oulu*			*Helsinki*			*Finland*		
**Year**	**Women**	**S**	**per** **100** **000**	**Women**	**S**	**per** **100** **000**	**Women**	**S**	**per** **100** **000**

1969	46 245	5	10.81	333 502	43	12.89	2 384 060	246	10.32
1970	45 656	4	8.76	325 034	58	17.84	2 378 351	220	9.25
1971	46 474	5	10.76	330 205	58	17.57	2 391 875	222	9.28
1972	47 633	5	10.50	333 507	57	17.09	2 404 350	239	9.94
1973	48 302	6	12.42	335 687	60	17.87	2 416 619	249	10.30
1974	49 272	11	22.33	337 470	47	13.93	2 428 572	255	10.50
1975	50 132	5	9.97	337 570	72	21.33	2 438 377	254	10.42
1976	50 410	12	23.81	336 980	66	19.59	2 444 444	253	10.35
1977	50 691	8	15.78	335 057	54	16.12	2 451 299	258	10.53
1978	50 964	11	21.58	334 547	53	15.84	2 457 298	237	9.65
1979	51 188	6	11.72	334 981	60	17.91	2 464 508	242	9.82
1980	51 582	4	7.76	335 630	63	18.77	2 472 935	264	10.68
1981	52 237	1	1.91	336 511	56	16.64	2 484 677	239	9.62
1982	53 059	10	18.85	338 116	59	17.45	2 498 846	267	10.69
1983	53 225	6	11.27	339 108	56	16.51	2 512 686	249	9.91
1984	53 443	3	5.61	340 162	54	15.88	2 524 520	253	10.02
1985	53 976	4	7.41	341 781	58	16.97	2 532 884	249	9.83
1986	54 349	12	22.08	343 576	53	15.43	2 539 778	287	11.30
1987	54 760	10	18.26	346 162	65	18.78	2 545 734	301	11.82
1988	55 125	9	16.33	346 880	62	17.87	2 552 991	296	11.59
1989	55 810	8	14.33	347 226	58	16.70	2 561 623	297	12.59
1990	56 294	8	14.21	348 913	90	25.79	2 572 274	324	11.60
1991	56 735	8	14.10	352 207	74	21.01	2 585 960	306	11.83
1992	57 391	6	10.46	354 429	77	21.73	2 597 700	297	11.43
1993	57 765	6	10.39	358 557	72	20.08	2 607 716	293	11.24
1994	58 781	12	20.42	363 774	77	21.17	2 617 105	307	11.73
1995	60 186	12	19.94	369 437	65	17.59	2 625 125	309	11.77
1996	61 447	8	13.02	373 663	69	18.47	2 631 724	282	10.72
1997	62 439	11	17.62	378 547	68	17.96	2 638 251	284	10.77
1998	63 454	11	17.34	382 880	57	14.89	2 643 571	268	10.14
1999	64 516	13	20.15	386 384	68	17.60	2 648 276	254	9.59
2000	66 149	11	16.63	389 425	66	16.95	2 651 774	292	11.01
2001	67 584	9	13.32	391 649	62	15.83	2 657 304	271	10.20
2002	68 297	11	16.11	392 485	48	12.23	2 661 379	275	10.33
2003	68 878	7	10.16	393 035	55	13.99	2 666 839	261	9.79

The yearly female population, number of suicides(S), and suicide mortality for women in Oulu, Helsinki, and Finland from 1969 to 2003.

**Figure 1:**
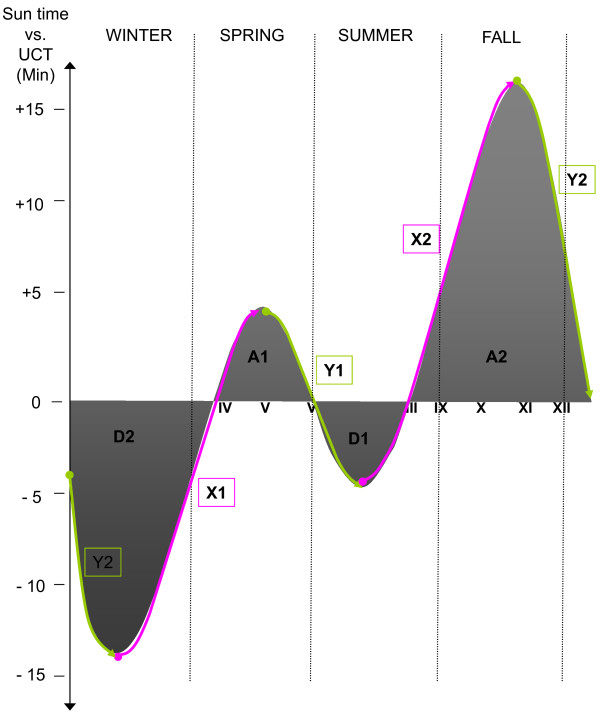
**Periods according to time of equation and astronomical seasons**. During X1(February 11-May 04) and X2 (July 26-November 03) (marked with pink lines) sun time is accelerating, and during Y1 (May 15-July25) and Y2 (November 04-February 10) (marked with green lines) it is decelerating compared with the coordinated universal time (UCT). During A1 (April 15-June13) and A2 (September 01-December 25) sun time stays ahead and during D1 (June 14-August 31) and D2 (December 26-April 14) it stays behind the UCT. Astronomical seasons are separated with dotted vertical lines. During astronomical spring and summer daylight exceeds darkness, and vice versa during astronomical fall and winter darkness exceeds daylight in Finland. Y-axis on the left side presents the time difference (in minutes) that sun time deviates from the UCT.

The nominal calendar year was split into twelve periods according to these two phenomena; first into four astronomical seasons, which are determined by spring and fall equinoxes and summer and winter solstices (for the definition, see http://asa.nao.rl.ac.uk/), and second into eight periods, by the equation of time (for the definition, see http://www.nmm.ac.uk/explore/astronomy-and-time/time-facts/the-equation-of-time), as follows (see also Figure 1). From February 11^th ^to May 14^th ^(hereafter marked as X1) and from July 26^th ^to November 3^rd ^(X2) ST goes fast compared with UCT and in between those periods, that is, from May 15^th ^to July 25^th ^(Y1) and from November 4^th ^to February 10^th ^(Y2) it goes slow. Furthermore, another categorization was made based on the equation of time separating periods when ST is either ahead or behind the UCT. In other words, ST is constantly ahead of the UCT, from April 15^th ^to June 13^th ^(A1) and from September 1^st ^to December 25^th ^(A2), and constantly behind the UCT, from June 14^th ^to August 31^st ^(D1), and from December 26^th ^to April 14^th ^(D2). Hence, ST deviates from UCT constantly and is maximally behind at February 11^th ^(approximately 14 minutes) and vice versa maximally ahead at November 3^rd ^(approximately 16 minutes). The Almanac Office at the University of Helsinki http://almanakka.helsinki.fi/ both provided the dates for the astronomical seasons and calculated the dates for the periods (X1, Y1, X2, Y2) of the equation of time, as well as the dates for the periods (A1, D1, A2, D2) through the whole study period.

To evaluate the effect of daily sunshine hours and temperature on suicide mortality, we focused on two cities on a similar longitude but with dissimilar photoperiod: first, Helsinki (60°9.7'N, 24°57.3'E), which is the capital of Finland in the south, and second, Oulu (65°1.0'N, 25°30.0'E), which is a central city of the northern part of the country, 600 km north from Helsinki. In Helsinki 5062 suicides were committed by men, and 2160 by women, whereas 903 by men and 278 by women in Oulu. The Finnish Meteorological Institute http://www.fmi.fi/ provided us with the daily data on sunshine hours and temperature, measured within the 25-km radius from these cities throughout the study period. For the day to day analysis, the daily sunshine, measured in minutes per day, (hereafter S) and the daily temperature, measured in degrees in Celsius and averaged as the daily mean value, (hereafter T) were compared with those on the previous day and changes were marked as (+) indicating an increase, and (-) indicating a decrease from the previous day. Thus, we ended with four types of days according to weather changes, coded as T+S+, T+S-, T-S+, and T-S-, concerning the data from Helsinki and Oulu regions.

In order to take into account the differences in the yearly population sizes within and between Helsinki and Oulu, the daily means of suicides were calculated into daily means of suicide mortality rates (suicides per 100,000), for men and women, per each year, and for both cities (Tables 1 and 2). Furthermore, in order to control for the different lengths of each period studied, and to avoid the bias of having dominance of certain type of weather changes within any period of the year, the daily mean of suicide mortalities (number of suicides per day, with 95% confidence intervals [CIs]) was calculated for each period in separate (Tables 3, 4, and 5).

**Table 3:** Astronomical seasons and men's (M) and women's (W) suicide mortality

	*Area*	*Selected Days*		*Astronomical season *			
				
				Winter	Spring	Summer	Fall
*M*	Finland	All		.099 .094-.103	.120 .115-.125	.117 .112-.122	.106 .101-.111
	Helsinki	All		.128 .120-.136	.138 .128-.148	.135 .123-.147	.131 .122-.140
		T+	S+	*.123* *.099-.146*	*.135* *.122-.147*	*.139* *.122-.156*	*.137* *.120-.155*
			S-	*.113* *.092-.134*	*.144* *.125-.163*	*.142* *.120-.164*	*.115* *.099-.132*
		T-	S+	*.122* *.108-.136*	*.146* *.128-.163*	*.130* *.117-.143*	*.133* *.118-.147*
			S-	*.122* *.097-.146*	*.140* *.120-.161*	*.129* *.112-.146*	*.144* *.116-.172*
	Oulu	All		.119 .098-.140	.141 .123-.160	.159 .138-.180	.131 .113-.150
		T+	S+	*.107* *.050-.163*	*.134* *.103-.165*	*.162* *.112-.212*	*.152* *.077-.227*
			S-	*.097* *.059-.135*	*.126* *.082-.170*	*.175* *.132-.217*	*.147* *.101-.193*
		T-	S+	*.124* *.094-.154*	*.167* *.132-.203*	*.156* *.115-.197*	*.117* *.073-.161*
			S-	*.097* *.045-.148*	*.138* *.110-.166*	*.132* *.086-.178*	*.144* *.083-.205*
*W*	Finland	All		.025 .023-.027	.031 .030-.032	.029 .028-.031	.028 .027-030
	Helsinki	All		.046 .042-.050	.050 .045-.054	.049 .045-.054	.048 .043-.053
		T+	S+	*.038* *.028-.048*	*.056* *.046-.065*	*.049* *.039-.058*	*.056* *.043-.069*
			S-	*.051* *.033-.069*	*.049* *.041-.056*	*.046* *.034-.058*	*.052* *.042-.062*
		T-	S+	*.042* *.036-.049*	*.046* *.034-.057*	*.049* *.040-.057*	*.049* *.039-.059*
			S-	*.052* *.039-.065*	*.052* *.042-.062*	*.051* *.041-.061*	*.045* *.031-.059*
	Oulu	All		.036 .029-.044	.040 .030-.049	.042 .032-.051	.039 .029-.048
		T+	S+	*.032* *.006-.058*	*.031* *.016-.046*	*.042* *.023-.061*	*.049* *.013-.086*
			S-	*.037* *.015-.058*	*.030* *.015-.043*	*.047* *.020-.074*	*.020* *.005-.036*
		T-	S+	*.041* *.019-.063*	*.051* *.028-.074*	*.037* *.019-.055*	*.043* *.019-.067*
			S-	*.028* *.000-.055*	*.045* *.020-.069*	*.042* *.020-.063*	*.047* *.018-.076*

Daily mean of suicide mortality and confidence interval of the mean in aggregate over the years from 1969 to 2003 for men and women, during astronomical seasons in Finland, Helsinki and Oulu, and according to daily changes (+/-) in temperature (T) and sunshine hours(S) in Helsinki and Oulu.

Winter: 21.12-20.03

Spring: 21.03-20.06

Summer: 21.06-22.09

Fall: 23.09-20.12

**Table 4:** Accelerating and decelerating periods of the equation of time and men's (M) and women's (W) suicide mortality

	*Area*	*Selected Days*		*Periods of time of equation*			
				
				X1	Y1	X2	Y2
*M*	**Finland**	All		.109 .105-.114	**.124** **.118-.129**	.112 .107-.116	.101 .096-.106
	Helsinki	All		.131 .121-.141	.142 .130-.154	.130 .122-.139	.130 .120-.140
		T+	S+	*.139* *.116-.161*	*.149* *.131-.167*	*.122* *.097-.147*	*.124* *.097-.151*
			S-	*.151* *.123-.179*	*.145* *.119-.170*	*.124* *.103-.145*	*.110* *.088-.131*
		T-	S+	*.152* *.122-.182*	*.126* *.100-.153*	*.135* *.121-.150*	*.120* *.099-.141*
			S-	*.133* *.104-.161*	*.124* *.106-.142*	*.138* *.116-.160*	*.114* *.086-.142*
	Oulu	All		.133 .115-.150	.146 .124-.167	.150 .131-.169	.122 104-.140
		T+	S+	*.156* *.094-.218*	*.149* *.101-.198*	*.206* *.122-.290*	*.131* *.052-.210*
			S-	*.096* *.042-.150*	*.207* *.142-.272*	*.165* *.112-.217*	*.097* *.046-.148*
		T-	S+	*.199* *.138-.259*	*.140* *.090-.190*	*.138* *.092-.184*	*.115* *.068-.162*
			S-	*.123* *.065-.181*	*.158* *.114-.201*	*.175* *.107-.242*	*.056* *.008-.103*
**W**	**Finland**	All		.028 .027-.029	**.032** **.030-.033**	.030 .029-.031	.025 .024-.027
	Helsinki	All		.047 .043-.051	.053 .048-.058	.050 .045-.054	.043 .039-.048
		T+	S+	*.052* *.040-.064*	*.055* *.046-.065*	*.056* *.044-.068*	*.033* *.018-.048*
			S-	*.044* *.030-.057*	*.059* *.047-.072*	*.055* *.041-.069*	*.055* *.044-.067*
		T-	S+	*.051* *.032-.070*	*.049* *.036-.061*	*.049* *.040-.058*	*.036* *.028-.045*
			S-	*.051* *.034-.067*	*.046* *.034-.058*	*.048* *.037-.058*	*.045* *.025-.065*
	Oulu	All		.038 .029-.047	.045 .034-.057	.035 .027-.042	.038 .029-.047
		T+	S+ S-	*.023* *.005-.042*	*.039* *.017-.060*	*.044* *.014-.074*	*-*
				*.035* *.012-.058*	*.053* *.018-.087*	*.015* *.001-.029*	*.026* *.002-.050*
		T-	S+ S-	*.062* *.028-.096*	*.030* *.006-.054*	*.042* *.024-.061*	*.036* *.013-.059*
				*.030* *.003-.058*	*.060* *028-.091*	*.032* *.010-.054*	*-*

Daily mean of suicide mortality and confidence interval of the mean in aggregate over the years from 1969 to 2003 for men and women, during accelerating (X1, X2) and decelerating (Y1, Y2) periods of the equation of time in Finland, Helsinki and Oulu, and according to daily changes (+/-) in temperature (T) and sunshine hours(S) in Helsinki and Oulu.

X1: 11.02-14.05

Y1: 15.05-25.07

X2: 26.07-03.11

Y2: 04.11-10.2

**Table 5:** Advanced and delayed periods of the equation of time and men's (M) and women's (W) suicide mortality

	*Area*	*Selected days*		*Periods of time of equation*			
				
				D2	A1	D1	A2
*M*	Finland	All		.102 .098-.107	.125 .120-.130	.118 .112-.123	.107 .102-.111
	Helsinki	All		.128 .121-.136	.146 .134-.158	.135 .122-.147	.130 .122-.139
		T+	S+	*.125* *.103-.146*	*.142* *.129-.154*	*.141* *.123-.159*	*.128* *.108-.148*
			S-	*.123* *.102-.143*	*.142* *.119-.164*	*.145* *.121-.169*	*.116* *.100-.131*
		T-	S+	*.124* *.111-.136*	*.162* *.137-.187*	*.124* *.108-.141*	*.134* *.121-.146*
			S-	*.123* *.103-.144*	*.147* *.124-.171*	*.126* *.110-.142*	*.141* *.118-.164*
	Oulu	All		.125 .107-.144	.145 .123-.167	.148 .128-.168	.139 .119-.159
		T+	S+	*.099* *.059-.139*	*.159* *.123-.196*	*.137* *.088-.185*	*.186* *.121-.250*
			S-	*.105* *.065-.145*	*.129* *.080-.178*	*.177* *.129-.225*	*.150* *.104-.196*
		T-	S+	*.140* *.112-.168*	*.155* *.103-.208*	*.146* *.095-.197*	*.129* *.090-.168*
			S-	*.109* *.062-.157*	*.137* *.097-.176*	*.122* *.079-.165*	*.149* *.091-.206*
W	Finland	All		.026 .024-.027	.032 .030-.034	.030 .028-.031	.028 .027-.030
	Helsinki	All		.046 .042-.049	.051 .045-.056	.051 .046-.056	.048 .043-.053
		T+	S+	*.043* *.034-.053*	*.058* *.047-.069*	*.048* *.039-.057*	*.056* *.047-.065*
			S-	*.050* *.033-.067*	*.047* *.033-.062*	*.049* *.038-.061*	*.052* *.042-.062*
		T-	S+	*.041* *.034-.048*	*.051* *.036-.066*	*.050* *.040-.061*	*.048* *.040-.056*
			S-	*.048* *.039-.058*	*.057* *.044-.069*	*.051* *.040-.062*	*.048* *.037-.059*
	Oulu	All		.036 .029-.044	.043 .032-.054	.041 .030-.053	.038 .031-.048
		T+	S+	*.037* *.016-.059*	*.031* *.013-.050*	*.036* *.017-.054*	*.044* *.019-.069*
			S-	*.033* *.016-.049*	*.033* *.013-.054*	*.058* *.024-.092*	*.017* *.005-.030*
		T-	S+	*.045* *.026-.063*	*.047* *.020-.075*	*.040* *.014-.066*	*.040* *.022-.058*
			S-	*.025* *.002-.048*	*.053* *.021-.085*	*.041* *.019-.064*	*.039* *.020-.057*

Daily mean of suicide mortality and confidence interval of the mean, in aggregate over the years from 1969 to 2003 for men and women, during advanced (A1, A2) and delayed (D1, D2) periods of the equation of time in Finland, Helsinki and Oulu, and according to daily changes (+/-) in temperature (T) and sunshine hours(S) in Helsinki and Oulu.

A1: 15.04-13.06

D1: 14.06-31.08

A2: 01.09-25.12

D2: 26.12-14.04

Finally, to rule out a potential confounder, we analyzed whether daylight saving time (hereafter DST) had any effect on the suicide mortality. DST was introduced in Finland 1981. From 1981 to 1994 DST lasted from the end of March until the end of September (hereafter DST1), and since 1995 DST has been in use from the end of March until the end of October, as in most parts of Europe (hereafter DST2). We calculated suicide mortality rates during one month period before, and after the transitions into and out of DST, separately for the years 1981 to 1994 (DST1) and years 1995 to 2003 (DST2), for which the suicide mortality rates of the corresponding periods during the years 1969 to 1980 were used as controls (Tables 6, 7, 8, 9,10, and 11).

**Table 6:** Men: Daily mean of suicide mortality, and switching into daylight saving time in spring.

	*Days*	1969-80*				1981-2003			
		
		-1 m		+1 m		-1 m		+1 m	
		M	CI	m	CI	m	CI	m	CI
Finland	All	.097	.089-.106	.111	.102-.121	.104	.097-.111	.117	.110-.124
Helsinki	All	.114	.096-.133	.130	.114-.146	.135	.120-.150	.137	.118-.155
	T+S+	*.120*	*.061-.180*	*.160*	*.100-.219*	*.129*	*.084-.173*	*.140*	*.102-.177*
	T+S-	*.100*	*.054-.146*	*.156*	*.100-.212*	*.128*	*.084-.171*	*.181*	*.143-.220*
	T-S+	*.123*	*.090-.156*	*.102*	*.067-.137*	*.139*	*.108-.171*	*.142*	*.100-.184*
	T-S-	*.144*	*.044-.243*	*.127*	*.061-.194*	*.180*	*.114-.246*	*.108*	*.080-.136*
Oulu	All	.113	.064-.162	.114	.060-.168	.121	.092-.150	.158	.117-.199
	T+S+	*-*	*-*	*-*	*-*	*-*	*-*	*.110*	*.042-.179*
	T+S-	*-*	*-*	*-*	*-*	*.145*	*.060-.229*	*.146*	*.055-.237*
	T-S+	*.123*	*.037-.208*	*.152*	*.013-.291*	*.104*	*.038-.171*	*.21*	*.139-.280*
	T-S-	*-*	*-*	*-*	*-*	*.065*	*.002-.129*	*.138*	*.039-.237*

Daily mean of suicide mortality during one month period before (-1 m), and after (+1 m) the switch into daylight saving time.

* = DST was not in use in Finland

m = daily mean of suicide mortality

CI = confidence interval of the mean

**Table 7:** Women: Daily mean of suicide mortality and switching into daylight saving time in spring.

*Location*	*Days*	1969-80 *				*1981-2003(DST1 + DST2)*			
		
		-1 m		+1 m		-1 m		+1 m	
		m	CI	m	CI	m	CI	m	CI
Finland	All	.024	.020-.028	.026	.023-.029	.027	.024-.030	.030	.027-.033
Helsinki	All	.055	.048-.062	.043	.031-.055	.043	.034-.052	.049	.040-.059
	T+S+	*.042*	*.014-.071*	*.052*	*.020-.083*	*.047*	*.019-.076*	*.055*	*.035-.076*
	T+S-	*.054*	*.028-.080*	*.031*	*.010-.052*	*.024*	*.012-.037*	*.046*	*.030-.062*
	T-S+	*.061*	*.041-.081*	*.029*	*.004-.053*	*.040*	*.022-.057*	*.065*	*.028-.101*
	T-S-	*.052*	*.018-.087*	*-*	*-*	*.049*	*.020-.079*	*.044*	*.024-.065*
Oulu	All	.033	.006-.059	.048	.016-.080	.048	.030-.066	.030	.009-.050
	T+S+	-	-	-	-	-	-	-	-
	T+S-	-	-	-	-	*.053*	*.013-.094*	-	-
	T-S+	-	-	-	-	*.067*	*.018-.117*	-	-
	T-S-	-	-	-	-	-	-	-	-

Daily mean of suicide mortality during one month period before (-1 m), and after (+1 m) the switch into daylight saving time.

* = DST was not in use in Finland

m = daily mean of suicide mortality

CI = confidence interval of the mean

**Table 8:** Men: Daily mean of suicide mortality, and switching away from daylight saving time in fall.

*Location*	*Days*	1969-80*				*1981-94 (DST1)*			
		
		-1 m		+1 m		-1 m		+1 m	
		m	CI	m	CI	m	CI	m	CI
Finland	All	.111	.099-.122	.101	.094-.108	.119	.109-.128	.120	.112-.128
Helsinki	All	.141	.106-.177	.120	.104-.137	.139	.125-.153	.140	.122-.158
	T+S+	*.151*	*.064-.238*	*.115*	*.078-.151*	*.091*	*.039-.143*	*.163*	*.107-.219*
	T+S-	*.156*	*.076-.236*	*.107*	*.048-.166*	*.124*	*.087-.161*	*.123*	*.079-.167*
	T-S+	*.137*	*.089-.185*	*.132*	*.083-.182*	*.156*	*.115-.197*	*.144*	*.107-.180*
	T-S-	*.138*	*.087-.189*	*.147*	*.085-.208*	*.184*	*.125-.244*	*.155*	*.062-.248*
Oulu	All	.114	.060-.168	.125	.092-.158	.239	.165-.313	.121	.067-.175
	T+S+	*-*	*-*	*-*	*-*	*.324*	*.113-.535*	*-*	*-*
	T+S-	*.100*	*.001-.200*	*.107*	*.002-.211*	*.256*	*.053-.458*	*.110*	*.000-.220*
	T-S+	*.189*	*.084-.295*	*.130*	*.024-.237*	*.182*	*.060-.303*	*.103*	*.029-.176*
	T-S-	*-*	*-*	*-*	*-*	*.267*	*.142-.392*	*.229*	*.065-.393*

Daily mean of suicide mortality during one month period before (-1 m), and after (+1 m) the switch away daylight saving time.

* = DST was not in use in Finland

m = daily mean of suicide mortality

CI = confidence interval of the mean

**Table 9:** Women: Daily mean of suicide mortality, and switching away from daylight saving time in fall.

*Location*	*Days*		1969-80 *				*1981-94 (DST1)*			
		
			-1 m		+1 m		-1 m		+1 m	
			m	CI	m	CI	m	CI	m	CI
Finland	All		.028	.025-.032	.029	.026-.033	.029	.024-.033	.035	.031-.039
Helsinki	All		.052	.039-.064	.048	.033-.062	.046	.030-.061	.064	.049-.078
	T+	S+	*.071*	*.045-.097*	*.039*	*.019-.058*	*-*	*-*	*.074*	*.040-.108*
		S-	*-*	*-*	*.069*	*.033-.104*	*.049*	*.015-.084*	*.058*	*.033-.083*
	T-	S+	*.049*	*.025-.073*	*.055*	*.021-.089*	*.052*	*.031-.073*	*.067*	*.038-.096*
		S-	*.070*	*.022-.118*	*.023*	*.001-.046*	*.032*	*.005-.059*	*.065*	*.026-.103*
Oulu	All		.033	.011-.055	.043	.006-.081	.020	.001-.040	.024	.001-.047
	T+S+		-	-	-	-	-	-	-	-
	T+S-		-	-	-	-	-	-	-	-
	T-S+		-	-	-	-	-	-	-	-
	T-S-		-	-	-	-	-	-	-	-

Daily mean of suicide mortality during one month period before (-1 m), and after (+1 m) the switch away daylight saving time.

* = DST was not in use in Finland

m = daily mean of suicide mortality

CI = confidence interval of the mean

**Table 10:** Men: Daily mean of suicide mortality, and switching away from daylight saving time in fall.

*Location *	*Days*	1969-80*				*1995-2003 (DST2)*			
		
		-1 m		+1 m		-1 m		+1 m	
		m	CI	m	CI	m	CI	m	CI
Finland	All	.101	.094-.108	.101	.093-.110	.105	.095-.115	.097	.088-.105
Helsinki	All	.126	.108-.145	.140	.119-.161	.118	.079-.157	.106	.090-.121
	T+S+	*.116*	*.073-.159*	*.181*	*.078-.285*	*.128*	*.045-.212*	*.080*	*.033-.128*
	T+S-	*.134*	*.085-.182*	*.072*	*.017-.127*	*.117*	*.059-.176*	*.109*	*.075-.142*
	T-S+	*.135*	*.079-.191*	*.177*	*.105-.249*	*.112*	*.071-.153*	*.093*	*.054-.131*
	T-S-	*.152*	*.072-.233*	*.204*	*.093-.314*	*.099*	*.035-.163*	*.104*	*.066-.142*
Oulu	All	.131	.089-.173	.106	.058-.154	.145	.096-.195	.169	.107-.230
	T+S+	*-*	*-*	*-*	*-*	*-*	*-*	*-*	*-*
	T+S-	*.104*	*.003-.205*	*-*	*-*	*.200*	*.069-.332*	*-*	*-*
	T-S+	*.167*	*.050-.284*	*.085*	*.001-.169*	*-*	*-*	*-*	*-*
	T-S-	*-*	*-*	*-*	*-*	*.148*	*.006-.290*	*-*	*-*

Daily mean of suicide mortality during one month period before (-1 m), and after (+1 m) the switch away daylight saving time.

* = DST was not in use in Finland

m = daily mean of suicide mortality

CI = confidence interval of the mean

**Table 11:** Women: Daily mean of suicide mortality, and switching away from daylight saving time in fall.

*Location *	*Days*	1969-80 *				*1995-2003 (DST2)*			
		
		-1 m		+1 m		-1 m		+1 m	
		m	CI	m	CI	m	CI	m	CI
Finland	All	.029	.026-.032	.025	.022-.029	.032	.026-.038	.027	.022-.032
Helsinki	All	.044	.029-.058	.042	.033-.051	.054	.039-.070	.035	.026-.044
	T+S+	*.032*	*.011-.052*	*.058*	*.019-.097*	*.104*	*.050-.159*	*.053*	*.002-.104*
	T+S-	*.055*	*.027-.083*	*.074*	*.036-.112*	*.049*	*.015-.083*	-	-
	T-S+	*.046*	*.015-.076*	*.032*	*.010-.054*	*.034*	*.002-.067*	*.040*	*.016-.065*
	T-S-	*.026*	*.001-.051*	-	-	*.064*	*.030-.099*	-	-
Oulu	All	.038	.005-.072	.032	.010-.054	.027	.004-.051	.051	.012-.090
	T+S+	-	-	-	-	-	-	-	-
	T+S-	-	-	-	-	-	-	-	-
	T-S+	-	-	-	-	-	-	-	-
	T-S-	-	-	-	-	-	-	-	-

Daily mean of suicide mortality during one month period before (-1 m), and after (+1 m) the switch away daylight saving time.

* = DST was not in use in Finland

m = daily mean of suicide mortality

CI = confidence interval of the mean

The 95% CIs of the daily mean values, controlled for the length of a period of study and the male and female population sizes in a region of study, were used to evaluate the statistical significance, so that if they did not overlap with each other, it was judged to indicate a marked statistical significance.

## Results

In Finland, during the years 1969 to 2003, the daily mean of suicide mortality was at the highest, with a statistical significance, for both men (mean = .124, CI = .118-.129) and women (mean = .032, CI = .030-.033), during the period Y1, i.e. from May 14^th ^to July 25^th ^, as compared to the nationwide references (Table 4).

### Local photoperiod

The highest daily mean of suicide mortality seem to have emerged later in Oulu compared with Helsinki, but only for men. Therefore, the results of men are reported here in more detail. The daily mean of suicide mortality was at the highest during the period Y1 in Helsinki (mean = .142, CI = .130-.154, Table 4), but during the period X2 i.e. from July 26^th ^to November 3^rd ^in Oulu (mean = .150, CI = .131-.169, Table 4). The same postponed pattern was found also when the time pattern of suicide mortality was evaluated by seasons. The daily mean of suicide mortality was highest in Helsinki during spring (mean = .138, CI = .128-.148), but during summer in Oulu (mean = .159, CI = .138-.180). Furthermore, a similar postponed pattern was seen from A1 (Helsinki) to D1 (Oulu) periods (Table 5). However, these results did not reach statistical significance.

### Local daily weather changes

For men, the days with T+S+ seem to have had the highest daily mean of suicide mortality both in Helsinki, during the period Y1 (mean = .149, CI = .131-.167), and in Oulu, during the period X2 (mean = .206, CI = .122-.290), which were the most "dangerous" periods in these cities. However, when estimated by the 95% confidence intervals, there was no statistical difference in the variation of means of suicide mortality between the four types of weather changes. The daily mean of suicide mortality in Helsinki and Oulu, however, do exceed the nationwide daily means of suicide mortality (mean = .124 for Y1 in Finland, and mean = .112 for X2 in Finland), as do all the underlined values for different types of weather changes in Helsinki and Oulu compared with each period at issue in Tables 3, 4, and 5.

### Daylight saving time

The use or timing of daylight saving time did not have a significant effect on the suicide mortality (Tables 6, 7, 8, 9, 10, and 11).

## Discussion

### Nationwide results

Our key finding of statistical significance demonstrates the increased suicide mortality on nationwide level in Finland during the period from May 14^th ^to July 25^th^. This 76-day period covers symmetrically both sides of summer solstice (Figure 1). During this period there is only 1 to 4 hours of darkness during the night in Helsinki but no darkness at all in Oulu. For the photoperiod dynamics in these locations, see http://www.gaisma.com/en/location/helsinki.html and http://www.gaisma.com/en/location/oulu.html, whose sunrise and sunset calculations are based on the algorithms displayed on National Oceanic and Atmospheric Administration Surface Radiation Research Branch web site at http://esrl.noaa.gov/gmd/grad/solcalc/calcdetails.html, and e.g. for latitudes less than 72° north and south, accuracy is approximately one minute. It is of note here that the photoperiod in Finland due to its time zone is asymmetrical throughout the year, the period of daylight being always shorter for the a.m. hours than it is for the p.m. hours. This phenomenon influences the mechanisms that decode the duration of the melatonin signal in the melatonin-target tissues.

From the circadian-clock point of view, this period (May 14^th ^to July 25^th^) is a challenge to alignment of the circadian rhythms with the sleep-wake cycle, and it resembles "the critical spring photoperiodic window" on intermediate to long days that has been characterized in sheep [37]. Some possible biological mechanisms for our current finding are briefly discussed in the following. The very long day (20 to 24 hours of daylight) might challenge the network within the circadian pacemaker that is comprised of the so-called evening and morning active cells, and that takes part in the seasonal adaptation in diurnal animals such as fruit flies [38,39] and sheep [37,40-42]. If this holds for humans as well, it is not known at the moment. If it does, it could mean that, when day lengths approximate fall and winter, the morning active cells dominate the circadian output, e.g. the sleep-wake behavior. This dominance of hierarchy is gradually transferred to the evening active cells as the days get longer in spring [38,39], the coincidence effect of the morning and evening active cells disappearing when the melatonin signal duration becomes insufficient to sensitize adenylate cyclase and to support a peak expression of the morning-active cells [37]. Interestingly, the speeding up of the evening active cells (e.g. by sunshine) makes the morning active cells run faster in long (summer) but not in short (winter) days [38]. In Finland, which is located at high to temperate latitudes with the light-dark transitions being most rapid around equinoxes, the asymmetrical photoperiod possibly favors the evening-active cells, and produces pronounced melatonin-dependent effects on gene expression during spring and fall. Whether such "locked morning active cells" contribute to the peak in deaths from suicide in spring in particular is not known. However, CRY2 and PER2 genetic variants, which might influence the evening and morning signals from the circadian pacemaker system, associate with depression vulnerability [43,44] in humans. Therefore, depressed individuals in particular might suffer from entrainment errors during periods that challenge the circadian pacemaker and predispose to circadian misalignment.

### Local daily weather changes

The complexity of the circadian pacemaker system suggests that signals other than the seasonal changes in photoperiod, such as temporary variations in local weather conditions, are likely to play a role in the entrainment process [35,45]. Our finding of the later suicide peak in the northern area of study, Oulu-region, supports this. However, the daily mean of suicide mortality was almost as high also during the Y1 period in Oulu, as in Helsinki.

Hereafter we discuss the potential influence of daily weather changes for the suicide mortality in Helsinki and Oulu during the peak periods.

During the most dangerous periods, Y1 in Helsinki and X2 in Oulu, days with T+S+ seemed to be the worst for suicide mortality. From the circadian point of view the long daylight combined with the daily increase in ambient temperature and sunshine hours (T+S+) may have further phase advanced the circadian rhythm of the male suicide victims. An increase in sunshine hours and exposure to light may accelerate and advance the phase of the principal circadian clock, but an increase in ambient temperature and exposure to heat may have a similar effect [46]. The peaks of suicides have associated with ambient temperature in earlier studies [47-49], but so far, to our knowledge, the role of the circadian clocks has not been addressed.

Many lines of evidence suggest that abnormalities in the thermoregulatory processes are common among the depressed and therefore may cause or maintain the circadian misalignment. Patients with a major depressive episode tend to have elevated body temperature throughout the night, not during the day, and a phase advance of the circadian rhythm of core body temperature [18]. As hot nights might advance the phase of the circadian clock [50], and nocturnal body temperature during rapid-eye-movement sleep is influenced by hot, not cold, ambience [51], the dynamics of nocturnal temperatures might contribute to the advanced and rather fixed phase positions of circadian rhythms in major depressive episodes. In addition, sleep abnormalities, characteristically excessive rapid-eye-movement sleep at the cost of slow-wave sleep [17], are likely to give an abnormal (accelerating) feedback to the principal circadian pacemaker [26]. Further, during winter the duration of rapid-eye-movement sleep per night tends to increase [52], giving no support to deceleration and thereby favoring the desynchronization that may result in lowered mood and the subsequent increase in risk of suicide.

Daily fluctuations in temperature may play a part in the timing of suicides, either in combination with the long day length, or possibly also as a separate stressor. Studies concerning the over-activity in the functions of brown adipose tissue among the depressed [53] are most interesting in this respect, since the over-activity of brown adipose tissue may lead to reduced adaptation to rapid changes in ambient temperature that are typical during spring and fall. Once being activated, brown adipose tissue does not become quiescent easily [54], and if having been over-activated, it may through the thermoregulatory defect lead to disruption of the sleep-wake cycle and appetite control, and lead to early morning awakenings and loss of weight of the affected individual. Whether this kind of "vernalization failure" characterizes a suicide process and contributes to a mortality peak year after year is not known, but needs experimental data for analysis. However, in line with this background, for the Y1 period the daily mean of suicide mortality of men was at its lowest during the days of T-S- in Helsinki (mean = .124, CI = .106-.142, Table 4) and during the days of T-S+ in Oulu (mean = .140, CI = .090-.190, Table 4), suggesting that T- is a common nominator for the "safer" weather changes in both locations. T-S+ days were the "safest" also during the X2 period in Oulu. The daily decrease in temperature could therefore serve as a protective change during otherwise warm season. However, as the daily means did not differ significantly between the four types of weather changes, this is somewhat speculative thus far.

### Limitations

Our limitation here is that we did not have the diagnostic information of the suicide victims, and that we demonstrate associations only, which do not necessarily tell anything about causality. Another limitation is that we did not have access to a suitable method, e.g. molecular-timetable methods [55] to be applied to a range of tissues, such as the brain and brown adipose tissue, from autopsy studies, to be able to analyze a mechanism of action and thereby to demonstrate a potential link between abnormalities in the circadian pacemaker system and death from suicide. On the other hand, our strengths include the nationwide sample of suicides for a long period of time, from a country with a high suicide mortality rate.

## Conclusions

Our main findings here are that suicide mortality is higher during summer months and that daily changes in sunshine and ambient temperature are likely to modify the suicide mortality. Our findings presented herein now wait for tests by others in independent materials and is thus open to replication and the subsequent verification or falsification of the hypothesis. Some experimental data would be urgently needed for explanation of the mechanisms of action that take place in the brain of depressed patients and predispose them to suicide within those particular periods of time that we identified here. Suicide is a long process, whereas the timing of death from suicide appears far from random. In Finland from 1969 to2003 suicide mortality was elevated from May 15^th ^to July 25^th^. This phenomenon should be considered also in clinical practice, since it bears implications for suicide prevention.

## Competing interests

The authors declare that they have no competing interests.

## Authors' contributions

Authors TP and KS designed the study and wrote the protocol. Author JL conceived and took part in designing the study. Authors LH and TP managed the literature searches and analyses and author LH wrote the first draft of the manuscript. All authors contributed to and approved the final manuscript.
